# Randomized trial of one-hour sodium bicarbonate vs standard periprocedural saline hydration in chronic kidney disease patients undergoing cardiovascular contrast procedures

**DOI:** 10.1371/journal.pone.0189372

**Published:** 2018-02-08

**Authors:** Judith Kooiman, Jean-Paul P. M. de Vries, Jan Van der Heyden, Yvo W. J. Sijpkens, Paul R. M. van Dijkman, Jan J. Wever, Hans van Overhagen, Antonie C. Vahl, Nico Aarts, Iris J. A. M. Verberk-Jonkers, Harald F. H. Brulez, Jaap F. Hamming, Aart J. van der Molen, Suzanne C. Cannegieter, Hein Putter, Wilbert B. van den Hout, Inci Kilicsoy, Ton J. Rabelink, Menno V. Huisman

**Affiliations:** 1 Department of Thrombosis and Hemostasis, Leiden University Medical Center, Leiden, the Netherlands; 2 Department of Nephrology, Leiden University Medical Center, Leiden, The Netherlands; 3 Department of Vascular Surgery, St. Antonius Hospital, Nieuwegein, the Netherlands; 4 Department of Cardiology, St. Antonius Hospital, Nieuwegein, the Netherlands; 5 Department of Internal Medicine, Bronovo Hospital, The Hague, the Netherlands; 6 Department of Cardiology, Bronovo Hospital, the Hague, the Netherlands; 7 Department of Vascular Surgery, Haga Teaching Hospital, The Hague, the Netherlands; 8 Department of Radiology, Haga Teaching Hospital, The Hague, the Netherlands; 9 Department of Vascular Surgery, Onze Lieve Vrouwe Gasthuis, Amsterdam, the Netherlands; 10 Department of Radiology, Bronovo Hospital, The Hague, the Netherlands; 11 Department of Nephrology, Maasstad Hospital, Rotterdam, the Netherlands; 12 Department of Nephrology, St. Lucas Andreas Hospital, Amsterdam, the Netherlands; 13 Department of Vascular Surgery, Leiden University Medical Center, Leiden, the Netherlands; 14 Department of Radiology, Leiden University Medical Center, Leiden, the Netherlands; 15 Department of Clinical Epidemiology, Leiden University Medical Center, Leiden, the Netherlands; 16 Department of Medical Statistics, Leiden University Medical Center, Leiden, the Netherlands; 17 Department of Medical Decision Making, Leiden University Medical Center, Leiden, the Netherlands; Medizinische Universitat Graz, AUSTRIA

## Abstract

**Background:**

Guidelines advise periprocedural saline hydration for prevention of contrast induced-acute kidney injury (CI-AKI). We analysed whether 1-hour sodium bicarbonate hydration administered solely prior to intra-arterial contrast exposure is non-inferior to standard periprocedural saline hydration in chronic kidney disease (CKD) patients undergoing elective cardiovascular diagnostic or interventional contrast procedures.

**Methods:**

We performed an open-label multicentre non-inferiority trial between 2011–2014. Patients were randomized to 1 hour pre-procedure sodium bicarbonate hydration (250 ml 1.4%, N = 168) or 4–12 hours saline hydration (1000 ml 0.9%, N = 165) prior to and following contrast administration (2000 ml of saline total). Primary outcome was the relative serum creatinine increase (%) 48–96 hours post contrast exposure. Secondary outcomes were: incidence of CI-AKI (serum creatinine increase>25% or >44μmol/L), recovery of renal function, the need for dialysis, and hospital costs within two months follow-up.

**Results:**

Mean relative creatinine increase was 3.1% (95%CI 0.9 to 5.2%) in the bicarbonate and 1.1% (95%CI -1.2 to 3.5%) in the saline arm, mean difference 1.9% (95%CI -1.2 to 5.1%, p-non-inferiority <0.001). CI-AKI occurred in 11 (6.7%) patients randomized to sodium bicarbonate and 12 (7.5%) to saline (p = 0.79). Renal function did not fully recover in 40.0% and 44.4% of CI-AKI patients, respectively (p = 0.84). No patient required dialysis. Mean costs for preventive hydration and clinical preparation for the contrast procedure were $1158 for sodium bicarbonate vs. $1561 for saline (p < 0.001).

**Conclusion:**

Short hydration with sodium bicarbonate prior to elective cardiovascular diagnostic or therapeutic contrast procedures is non-inferior to standard periprocedural saline hydration in CKD patients with respect to renal safety and results in considerable healthcare savings.

**Trial registration:**

Netherlands Trial Register (http://www.trialregister.nl/trialreg/index.asp), Nr NTR2699

## Introduction

Contrast induced-acute kidney injury (CI-AKI) is a common complication among patients undergoing cardiovascular diagnostic or interventional contrast procedures [1]. The development of CI-AKI is associated with morbidity, mortality, and a prolonged hospital stay [2–4]. Guidelines on the prevention of CI-AKI recommend the use of periprocedural intravenous saline (4–12 hours prior to and following contrast exposure) or sodium bicarbonate (1 hour prior to and 6 hours following contrast exposure) hydration in patients with chronic kidney disease (CKD), who are at particularly high risk of developing CI-AKI [5–7]. However, such use of CI-AKI preventive hydration is burdensome to patients and increases healthcare costs.

A previous randomized trial on the prevention of CI-AKI performed by our research group demonstrated the efficacy and safety of a 1-hour sodium bicarbonate regime administered solely prior to intravenous contrast enhanced-CT in patients with CKD [8]. The volume expansion of this short regime would prevent patients from being in a hypovolemic state at time of contrast administration, an important risk factor for CI-AKI. Yet, it is unclear whether these results can be adopted to CKD patients undergoing elective cardiovascular diagnostic or interventional procedures requiring intra-arterial contrast administration. Hence, the aim of this study was to assess whether 1-hour sodium bicarbonate hydration prior to contrast exposure is non-inferior to standard periprocedural saline hydration in this specific setting.

## Methods

We performed a multicenter randomized non-inferiority trial in one academic hospital, and seven non-academic teaching hospitals, see supplemental material (S1 Supplemental material**:** List of participating hospitals). Prior studies have shown sodium bicarbonate to be superior over saline hydration. However, as we strongly reduced the volume of sodium bicarbonate compared with these previous studies (who used bicarbonate hydration one hour prior to and six hours following contrast administration), we choose for a non-inferiority design for the comparison with saline hydration. In- and outpatients undergoing elective diagnostic or therapeutic cardiovascular procedures requiring intra-arterial contrast administration (i.e. peripheral percutaneous transluminal angiography, percutaneous coronary intervention, coronary angiography, endovascular aneurysm repair, angiography, or digital subtraction angiography) were screened for inclusion. We included patients 18 years or older with an estimated glomerular filtration rate (eGFR, calculated using the Modification of Diet in Renal Disease formula [9]) < 45 ml/min, or an eGFR 45–60 ml/min in combination with diabetes mellitus or at least two other risk factors for the development of CI-AKI (i.e. peripheral arterial disease, congestive heart failure, age > 75 years, anemia, use of diuretics or non-steroidal anti-inflammatory drugs) [10]. Patients were excluded if they were on dialysis treatment, received iodinated contrast media in the preceding seven days, currently had acute kidney injury, were pregnant, or had a documented allergy for iodinated contrast media.

The trial was approved by the Institutional Review Boards (main approval provided by the ethics committee of the Leiden University Medical Center, Leiden, the Netherlands) of the participating centers and performed according to the declaration of Helsinki. All patients provided written informed consent. Study outcomes were periodically reviewed by an independent data and safety monitoring board. The trial was registered at the Nederlands Trial Register (www.trialregister.nl), under number NTR2699.

### Randomization

Randomization was performed in a 1: 1 ratio using a computer generated allocation sequence using block randomization by a certified online program (https://www.msbi.nl/promise/), using an at random varying block size of 2, 4 or 6. The study had an open-label design. Randomization was stratified for hospital of inclusion, renal function (i.e. eGFR 0–20, 20–40, 40–60 ml/min) at time of randomization and whether a patient had been diagnosed with diabetes mellitus, as both severe chronic kidney disease (eGFR < 30 ml/min) and diabetes mellitus are important risk factors for the development of CI-AKI [1,11].

### Procedures

Patients were randomized to 1-hour pre-procedural intravenous hydration using 250 ml 1.4% sodium bicarbonate or periprocedural intravenous hydration with 0.9% saline, 1000 ml in 4–12 hours prior to and 1000 ml in 4–12 hours following contrast administration (total volume 2000 ml) [8]. Infusion rates for saline hydration were adjusted to a patient’s cardiac condition based on the clinical judgment (symptoms or a history of congestive heart failure) of the treating physician. Contrast media use in the eight participating hospitals was according to clinical practice and included the use of Iobitridol (Xenetix, Guerbet, Aulnay-sous-Bois, France), Iodixanol (Visipaque, GE Healthcare, Chalfort St. Giles, UK)), and Iopromide (Ultravist, Bayer Schering Pharma, Berlin, Germany) in concentrations of 270, 300, 320, and 370 mg I/ml. Patients did not receive other CI-AKI preventive treatments besides their randomized hydration regimen.

Serum and urine samples were collected at baseline (prior to hydration and contrast exposure), 4–6 and 48–96 hours following the contrast procedure in all patients, and after two months in patients developing CI-AKI. All samples were shipped to the laboratory of the Leiden University Medical Center after trial completion for re-analysis of serum creatinine values (Roche Diagnostic analyzers, Mannheim, Germany) and assessment of urinary pH-values. Urinary pH-values were measured to determine whether the use of sodium bicarbonate had alkalinized urine.

### Outcomes

Primary outcome of the study was the relative increase in serum creatinine (%) measured once in the 48–96 hours following contrast exposure compared with baseline [8]. Secondary outcomes were the incidence of CI-AKI (at 48–96 hours following contrast exposure), pulmonary oedema, recovery of renal function (i.e. no longer fulfilling the criteria of CI-AKI compared with baseline), a need for dialysis, re-hospitalization, and outpatient visits within two months follow-up.

Patients diagnosed with CI-AKI (defined as a serum creatinine increase > 25% or > 44 μmol/L compared with baseline [1]) based on serum creatinine values measured at the hospital of inclusion were asked to return to the outpatient clinic two months after contrast exposure to assess whether their renal function had recovered. Patients in whom the diagnosis of CI-AKI was not made based on the creatinine values measured at the hospital of inclusion but who did fulfill the criteria of CI-AKI grounded by the creatinine values quantified after trial completion were lost to follow-up for prospective assessment of the endpoint of recovery of renal function. For those patients, medical charts were scrutinized retrospectively for serum creatinine values assessed in routine practice approximately two months following contrast administration.

In addition, the incidences of CI-AKI based on the AKI definitions of the AKI network (AKIN) criteria were calculated [12].

### Economic evaluation

To analyze whether the use of sodium bicarbonate results in healthcare savings, costs were estimated from a hospital perspective, with a 2-month time horizon, at the price level of 2015. We registered hospital (re)admissions (including length of stay), outpatient visits and visits to the emergency department that took place between randomisation and two months follow-up. Infusion fluids were valued using market prices and all other health care using standard prices, designed to reflect societal costs and to standardize economic evaluations [13]. Costs for preventive hydration and clinical preparation prior to the contrast procedure were calculated separately. These costs were defined as costs for the randomized infusion fluids ($6 for sodium bicarbonate, $4 for saline), day care or inpatient hospitalization on the day of the contrast procedure ($483 or $760, respectively), inpatient hospitalization on the day prior to the contrast procedure, and inpatient hospitalization on the day following the contrast procedure for those discharged on that particular day. Costs for contrast media and serum creatinine (or other laboratory) measurements were not taken into account. We used cost-effectiveness acceptability curves to relate the difference in healthcare costs to the difference in CI-AKI incidence (according to intention-to-treat, with multiple imputation for missing values on the occurrence of CI-AKI and one-sided unequal-variance *t*-tests). Acceptability curves show the probability that one strategy is cost-effectiveness compared to the other strategy (i.e. has a better net benefit NB = WTP × incidence − Costs), depending on the willingness to pay (WTP) to prevent one case of CI-AKI [14].

### Statistical analyses

Our study had a non-inferiority design. As a low relative serum creatinine increase in the sodium bicarbonate group was expected, the use of sodium bicarbonate was considered non-inferior to saline hydration if the mean serum creatinine increase in the sodium bicarbonate group was not more than 15% higher compared with the increase in patients treated with saline [8]. A difference in mean relative serum creatinine < 15% was considered clinically nonsignificant, as creatinine changes following contrast media administration are generally of a reversible nature. The sample size was calculated at 152 patients per study arm based on an expected difference in the mean serum creatinine increase of 5% with a standard deviation of 31% (α 0.025, β 0.800) [15]. Taking into account a loss to follow-up of 15%, our total target sample size comprised 346 patients.

Study outcomes were computed by an independent medical statistician blinded for randomization, using an intention-to-treat approach. The primary endpoint of an increase in serum creatinine was tested using an independent samples *t*-test. Additionally, an one-sided p-value for non-inferiority was calculated under the null hypothesis of equivalence by first calculating a Z-statistic given by z = (equivalence margin—point estimate)/standard error, and subsequently calculating the probability of a standard normal random variable exceeding Z. Here, equivalence margin = 15%, and point estimate and standard error are based on estimated probabilities of serum creatinine. For this specific analysis, a p-value below 0.025 was considered to be statistically significant. Secondary outcomes were tested for statistical differences between randomization groups using relative risks with corresponding two-sided 95% confidence intervals (CI). For both the primary endpoint and the endpoint of CI-AKI, patients lost to follow-up were excluded from the analysis (5 patients in each treatment arm). To test for different effects across subgroups at high risk of CI-AKI (i.e. those with eGFR < 30 ml/min, diabetes mellitus, or age > 75 years), defined by covariates, we used multiple linear (primary endpoint) or logistic (secondary endpoint of CI-AKI) regression analyses with randomization, covariate and randomization by covariate interaction. Calculations were performed using SPSS version 20.0 (IBM Corp, Armonk, New York, USA).

## Results

**Fig 1:**
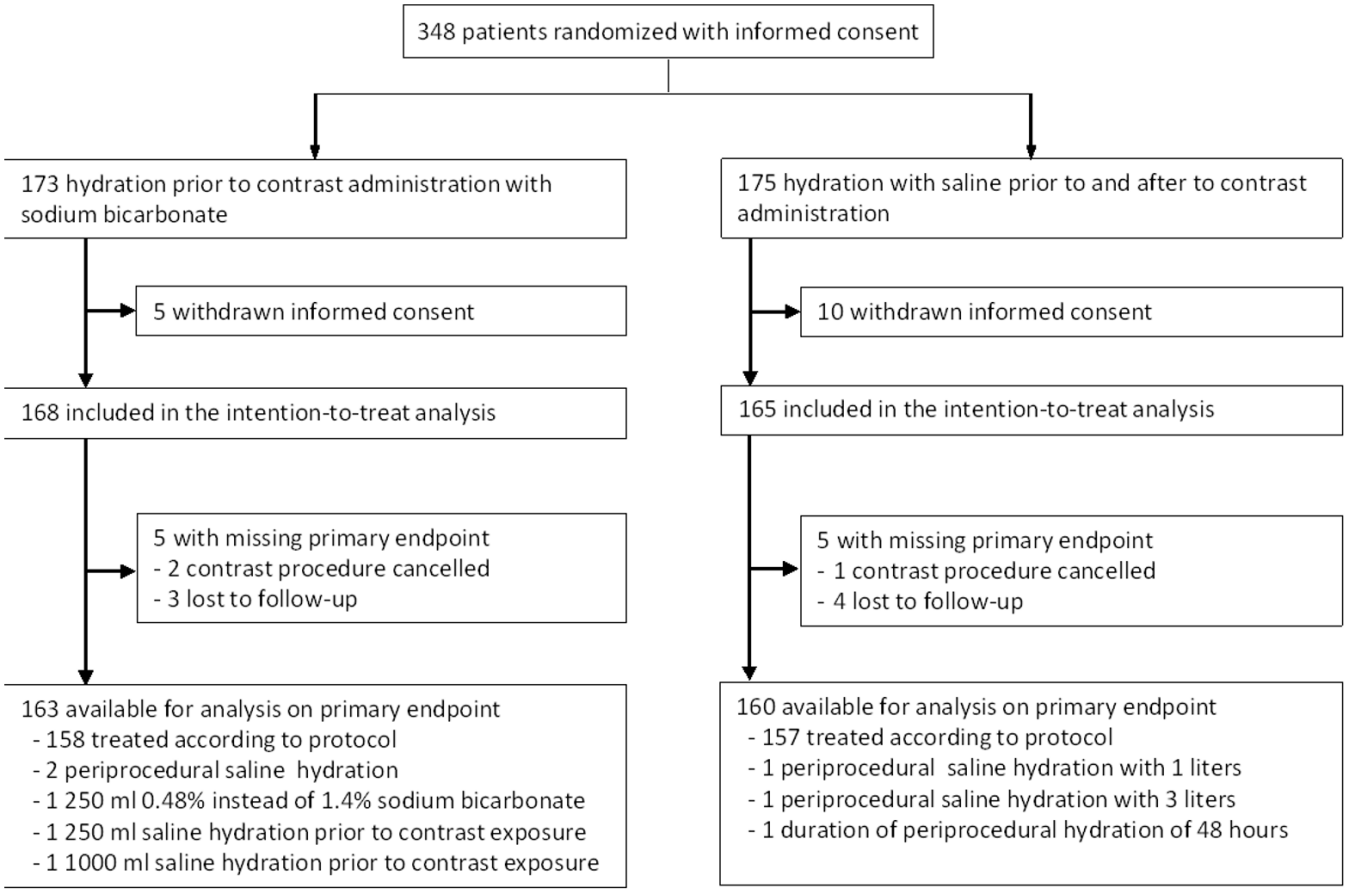
Trial profile.

We included and randomized 348 patients between 2011 and 2014, of whom 15 (4.3%) withdrew consent after randomization. As a result, the intention-to-treat population consisted of 333 patients; 168 randomized to sodium bicarbonate and 165 to saline hydration. Patient characteristics at baseline were well balanced between randomization arms, except for an imbalance in type of contrast procedure. Patients in the sodium bicarbonate group were less likely to undergo percutaneous transluminal angiography, yet more frequently underwent endovascular aneurysm repair (Table 1). Protocol violation occurred in 8 (2.4%) patients (Fig 1). In addition, the contrast procedure, and consequently hydration, was cancelled in 2 patients in the sodium bicarbonate and 1 patient in the saline group. All other patients received and completed the study mandated treatment, except for one patient in the saline arm in whom hydration was prematurely stopped because of signs of pulmonary oedema.

**Table 1:** Patient and procedure characteristics.

	Sodium bicarbonate (N = 168)	Saline (N = 165)
Mean age, years	73.0 (9.2)	72.5 (8.8)
Sex, male	105 (62.5%)	110 (66.7%)
Outpatients	159 (94.6%)	153 (92.7%)
Mean BMI	29.0 (11.4)	29.5 (21.7)
Mean eGFR	50.0 (14.8)	51.1 (16.7)
eGFR > 45 ml/min/1.73m2	107 (63.7)	103 (62.4)
eGFR 30–45 ml/min/1.73m2	47 (28.0)	46 (27.9)
eGFR < 30 ml/min/1.73m2	14 (8.3)	16 (9.7)
Mean systolic blood pressure	145.7 (22.1)	139.2 (21.1)
Mean diastolic blood pressure	76.7 (13.3)	74.2 (14.1)
Diabetes mellitus	65 (35.7%)	64 (38.8%)
Peripheral arterial disease	109 (64.9%)	119 (72.1%)
Coronary artery disease	92 (54.8%)	89 (53.9%)
Congestive heart failure	33 (19.6%)	22 (13.3%)
Primary renal or urological disease	107 (63.7%)	116 (70.3%)
Microalbuminuria*	12 (7.1%)	15 (9.1%)
Macroalbuminuria*	63 (37.5%)	67 (40.6%)
Medication		
Diuretics	102 (60.7%)	94 (57.0%)
ACE-inhibitors	76 (45.2%)	78 (47.3%)
Angiotensin II receptor blockers	45 (26.8%)	44 (26.7%)
Preprocedural stop of medication	11 (6.5%)	13 (7.9%)
Type of elective contrast procedure		
Angiography	9 (5.4%)	12 (7.3%)
DSA	4 (2.4%)	4 (2.4%)
PTA	88 (52.4%)	101 (61.2%)
EVAR	22 (13.1%)	7 (4.2%)
CAG	33 (19.6%)	30 (18.2%)
PCI	5 (3.0%)	3 (1.8%)
Other	5 (3.0%)	7 (4.2%)
Mean contrast volume in mL**	112.9 (44.9)	112.6 (48.1)
Mean iodine dose in grams	35.2 (14.1)	34.9 (15.6)
Median contrast volume/eGFR (2.5–97.5 percentiles)	2.3 (0.8–6.4)	2.2 (0.7–6.5)

Data are presented as n (%) or mean (SD) unless stated otherwise.

eGFR = estimated glomerular filtration rate. CKD = chronic kidney disease. DSA = digital subtraction angiography. PTA = percutaneous coronary intervention. EVAR = endovascular aneurism repair. CAG = coronary angiography. PCI = percutaneous coronary intervention.

* Microalbuminuria was defined as albumin-creatinine ratio 30–300 mg/g, macroalbuminuria as albumin-creatinine ratio > 300 mg/g.

** Missing in 34 and 40 patients, respectively.

### Study outcomes

**Fig 2:**
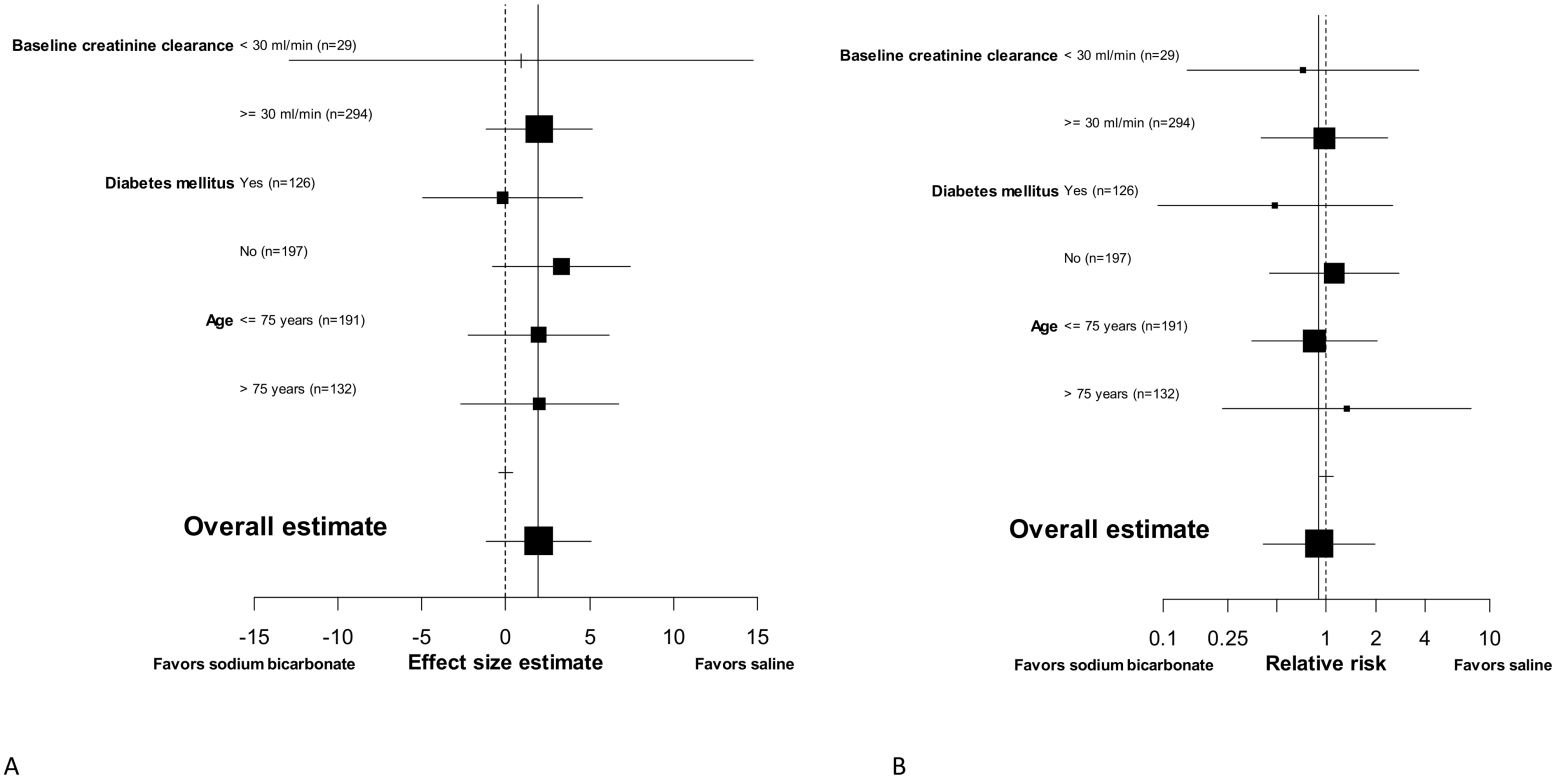
**A)** Subgroup analyses on the primary outcome of a relative increase in serum creatinine 48–96 hours post intra-arterial contrast administration. Effect size is calculated as the difference in the mean relative increase in serum creatinine between both randomisation groups. **B)** Subgroup analyses on the secondary outcome of risk of contrast-induced acute kidney injury, calculated as relative risk. The straight line indicates the point estimate of the entire study population and the dashed line indicates no effect. Baseline creatinine clearance was calculated using the MDRD-formula.

The primary endpoint of a relative increase in serum creatinine following contrast exposure compared with baseline and the secondary endpoint of CI-AKI were assessed in 323/333 (97.0%) patients. Mean relative increase in serum creatinine was 3.1% (95% CI 0.9 to 5.2%) in the sodium bicarbonate group and 1.1% (95% CI -1.2 to 3.5%) in patients treated with saline, for a mean difference of 1.9% (95% CI -1.2 to 5.1%, p-value for non-inferiority < 0.001), S1 Fig. The risk of CI-AKI was similar between randomization groups, 6.7% (11/163) in patients randomized to sodium bicarbonate and 7.5% (12/160) in patients randomized to saline hydration, relative risk 0.90 (95% CI 0.41–1.98). Risks of CI-AKI by randomization group according to the AKIN-criteria are presented in Table 2, and demonstrate a relative risk of 1.0 (95% CI 0.5–2.0) comparing sodium bicarbonate with saline hydration on AKIN stage 1. Of the 23 CI-AKI patients, 13 (56.5%) had a baseline eGFR > 45 ml/min, 5 (21.7%) between 30–45 ml/min, and 5 (21.7%) < 30 ml/min. The results on the primary endpoint and on the incidence of CI-AKI were homogenous for predefined subgroups of patients at high risk of CI-AKI, including those with severe CKD (Fig 2A and 2B), although 95% CI were wide. No significant interaction between randomization arms and the predefined subgroups was observed.

**Table 2:** Risks of acute kidney injury according to the acute kidney injury network criteria.

AKIN stage	Sodium bicarbonate		Saline		Relative risk (95% CI)
**I:** Increase > 26.5 umol/L or > 150% - 200% from baseline	16/163	(9.8%)	15/160	(9.4%)	1.0 (0.5–2.0)
**II:** Increase 200–300% from baseline	0/163	(0.0%)	0/160	(0.0%)	NA
**III:** Increase > 300%, or > 354 umol/L, or on RRT	0/163	(0.0%)	0/160	(0.0%)	NA

**Abbreviations:** RRT = renal replacement therapy, NA = not applicable

One CI-AKI patient in the sodium bicarbonate group and two CI-AKI patients randomized to saline were lost to follow-up for the endpoint of recovery of renal function. The diagnosis of CI-AKI in these three patients was based on serum creatinine values as measured after trial completion and not during their active trial participation, which precluded the prospective assessment of recovery of renal function. Unfortunately, renal function in those three patients had not been measured in the two months following the contrast procedure. In addition, one CI-AKI patient randomized to saline died within two weeks following contrast exposure of pneumonia. Renal function had not recovered to the pre procedure value within two months following the development of CI-AKI in 4/10 (40%) patients randomized to sodium bicarbonate vs. 4/9 (44%) patients in the saline arm with complete follow-up, relative risk 0.90 (95% CI 0.31–2.58) (S2 Fig). No patient required dialysis.

No patient randomized to sodium bicarbonate vs. 3 patients (1.9%) in the saline group developed pulmonary oedema, relative risk 0.14 (p-value 0.19). Among the 3 patients developing pulmonary oedema, hydration was prematurely stopped in 1. Two patients received furosemide treatment of whom 1 was admitted to the intensive care unit after successful resuscitation of a cardiac arrest due to volume overload.

Mean urinary pH was 6.0 (SD 0.9) at baseline and 6.9 (SD 1.0) at 4–6 hours following contrast exposure in the sodium bicarbonate group. For patients randomized to saline, these values were 5.8 (SD 0.7) and 6.4 (SD 0.9), respectively (p-value for difference in mean pH at 4–6 hours following contrast administration between randomization groups < 0.001).

### Healthcare cost perspective

Of the outpatients randomized to sodium bicarbonate 30/159 (18.9%) were treated in day care compared with 4/153 (2.6%) outpatients randomized to saline hydration. All other outpatients were admitted to the hospital on the day(s)prior to contrast administration or remained hospitalized for at least 24 hours following the contrast procedure (Table 3). Mean costs for preventive hydration and clinical preparation prior to the contrast procedure were $1158 for sodium bicarbonate vs. $1561 for saline, with a mean difference of $-403, (95% CI $-530 to -275), Table 3.

**Table 3:** Estimated hospital costs per patient between randomisation and two months follow-up.

	Sodium bicarbonate N = 168		Saline N = 165		Mean difference in $ (95% CI)
	Volume*	Mean cost in $ (SD)	Volume*	Mean cost in $ (SD)	
Costs related to contrast procedure					
- infusion fluids	0.96	4 (1)	0.96	3 (1)	1 (1 to 1)
- days prior to contrast exposure	0.52	394 (527)	0.93	705 (344)	-311 (-407 to -215)
- day of contrast exposure^1^	0.96	678 (178)	0.96	727 (149)	-48 (-84 to -13)
- day following contrast exposure^2^	0.11	82 (236)	0.17	126 (284)	-45 (-101 to 12)
- ICU days due to hydration complications†	0.00	0 (0)	0.01	38 (485)	-38 (-111 to 36)
- non-ICU days due to hydration complications†	0.0	0 (0)	0.12	93 (941)	-93 (-236 to 49)
- total costs related to the contrast procedure		***1158 (650)***		***1561 (522)***	***-403 (-530 to -275)***
Other hospitalization					
- following contrast exposure^3^	1.86	1414 (4458)	1.23	934 (3228)	480 (-363 to 1324)
- day care	0.14	69 (255)	0.17	80 (235)	-11 (-64 to 42)
- non-ICU, AKI^4^	0.00	0 (0)	0.08	61 (775)	-61 (-178 to 57)
- non-ICU, non-AKI^4^	1.97	1500 (4815)	2.11	1607 (4123)	-107 (-1077 to 864)
- ICU^4^	0.08	258 (2477)	0.04	133 (1698)	125 (-335 to 586)
Outpatient visits					
- emergency department	0.09	22 (75)	0.10	24 (83)	-2 (-19 to 15)
- nephrology	0.32	45 (119)	0.25	34 (100)	11 (-13 to 34)
- non-nephrology	2.84	395 (441)	3.28	456 (494)	-62 (-163 to 40)
*Total costs*		***4861 (7313)***		***5022 (6254)***	***-161 (-1634 to 1312)***

Abbreviations: ICU = intensive care unit, AKI = acute kidney injury

* Volumes represent percentage of patients or mean number of procedures, hospital days or visits

^†^ i.e. acute heart failure due to volume overload

^1^ Costs based on prices for either day-care or non-ICU days depending on duration of hospitalisation,

^2^ Only in those discharged on the day following the contrast procedure,

^3^ Excluding the day following contrast exposure in patients discharged on that following day,

^4^ Hospitalization not directly following contrast exposure

**Fig 3:**
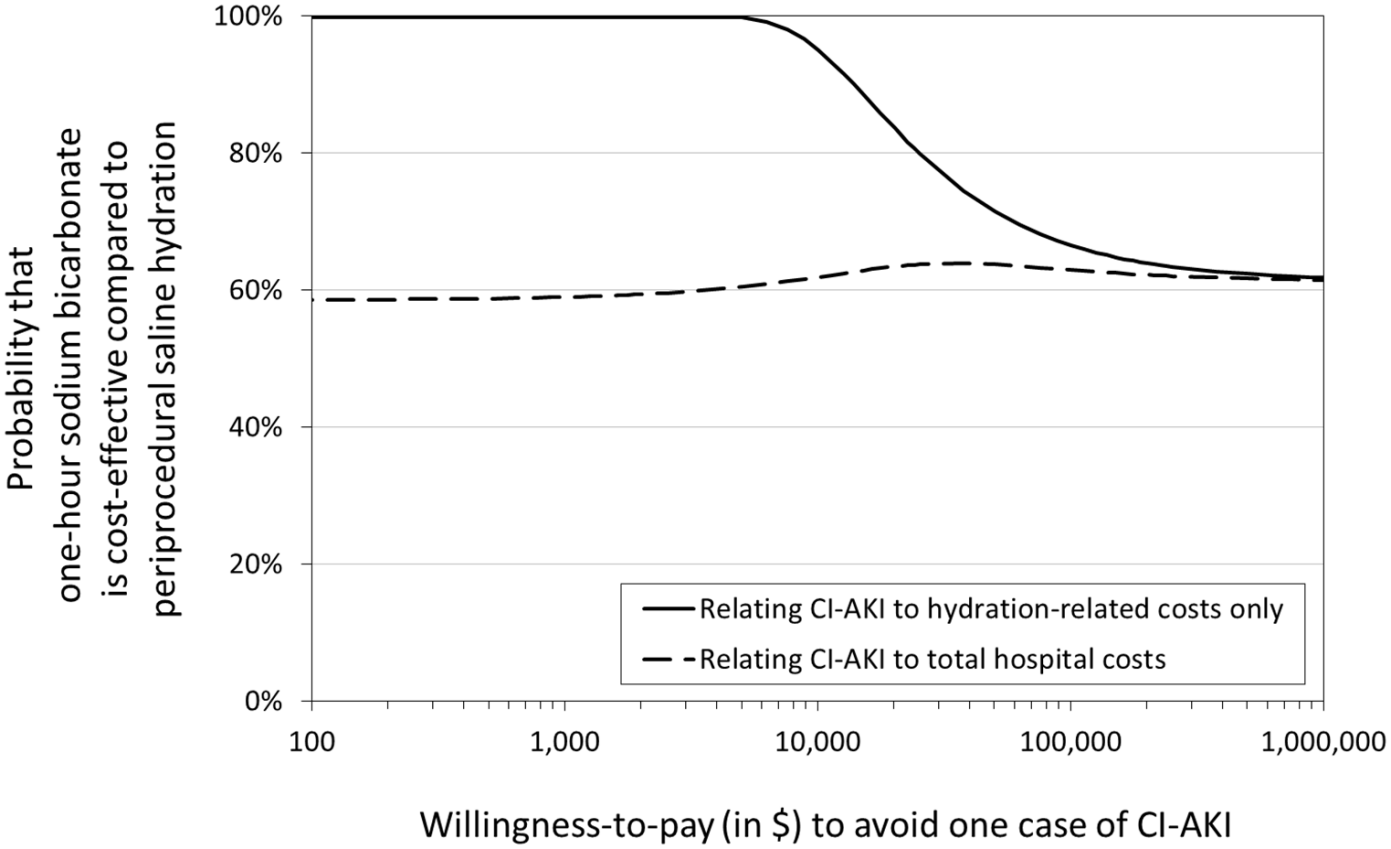
Whether a hydration strategy is cost-effective, depends on how much one is willing to pay (WTP) (in US dollars) to avoid one case of CI-AKI. This figure shows the probability that one-hour hydration with sodium bicarbonate prior to intra-arterial contrast administration is cost-effective compared with periprocedural saline hydration.

How much one is willing to pay (WTP) to prevent one case of CI-AKI determines whether a hydration strategy is considered cost-effective [13]. The probability that sodium bicarbonate hydration prior to contrast exposure is cost-effective compared with periprocedural saline hydration is shown in Fig 3. Costs and effectiveness in terms of incidences of CI-AKI are both non-significantly in favour of hydration with sodium bicarbonate (Table 3). Therefore, regardless of the WTP to avoid CI-AKI and taking all hospital costs into account, hydration with sodium bicarbonate is about 60% likely to be more cost-effective than standard saline hydration (Fig 3). Restriction to only the costs of hydration and clinical preparation for the contrast procedure, the estimated costs difference is larger and more certain. As a result, for a WTP of up to $10,000 to avoid one case of CI-AKI, the use of sodium bicarbonate is at least 90% likely to be more cost-effective (Fig 3).

## Discussion

The present results show that the use of a single administration of sodium bicarbonate hydration 1 hour prior to elective cardiovascular diagnostic or therapeutic procedures is non-inferior to periprocedural saline hydration in patients with CKD, in terms of a mean relative increase in serum creatinine. Second, the use of sodium bicarbonate instead of saline hydration results in a 30% reduction in healthcare cost, of more than $400 per patient. This cost reduction was mainly due to a 0.43 decrease in hospital days prior to contrast exposure. Therefore, the use of this brief sodium bicarbonate regimen is more likely to be cost-effective than standard periprocedural saline hydration.

Over the last decades, significant effort has focused on CI-AKI preventive measures in different patient settings [16–19]. Using this large body of data, most guidelines advice the use of either periprocedural saline hydration, which often results in a patients admission for two to three days, or periprocedural sodium bicarbonate hydration administered 1 hour prior to and 6 hours following contrast administration [1,20]. Although the use of periprocedural sodium bicarbonate instead of saline hydration shortens the duration of hospitalisation, the six hours of hydration following the contrast procedure often make it unfeasible to treat patients in a day care setting. Based on the findings of our study, the use of sodium bicarbonate can be reduced to a single bolus of 250 ml prior to contrast exposure, increasing the feasibility of day care treatment.

In addition, it should be emphasized that the development of AKI following contrast procedures is multifactorial and not restricted to contrast media use. Other etiologies such as shedding of cholesterol emboli into the renal vasculature, post procedural bleeding or hemodynamic instability resulting in acute tubular necrosis due to poor perfusion, or the use of nephrotoxic medication should also be taken into consideration [21,22]. Unfortunately, it is often difficult to differentiate between these causes of AKI as they all result in an increase in serum creatinine within days following the contrast procedure, frequently in absence of other symptoms.

With comparable efficacy of two hydration regimes, value-based care perspectives and patient convenience become of increased importance. In our study, the use of sodium bicarbonate was non-inferior to standard saline hydration, yet healthcare savings with the use of sodium bicarbonate were considerable. Additionally, the proportion of patients that can be treated in day care increased with the use of this brief sodium bicarbonate regime, improving patient convenience.

Another aspect that should be considered is safety. Although not statistically significant in our study, the use of periprocedural saline hydration was associated with pulmonary oedema, in one patient even leading to cardiac arrest and admission to the intensive care unit. This association between periprocedural saline hydration and acute heart failure has also been reported by other studies on the prevention of CI-AKI [8,18,23], while it has not (yet) been observed in patients treated with sodium bicarbonate, most likely due to the smaller amount of volume expansion [8,24].

Our study extends prior work in the field as it is the first trial to compare the use of a 1-hour pre-procedural sodium bicarbonate regime with periprocedural saline hydration in patients with CKD undergoing cardiovascular diagnostic or therapeutic procedures requiring intra-arterial contrast administration. Second, our study had a robust design, with few drop outs on the primary outcome. The results of our study were homogenous among the predefined subgroups of patients at high risk of CI-AKI. Moreover, the 7% risk of CI-AKI found in our study corresponds well with the incidence of CI-AKI following elective cardiovascular interventional or diagnostic contrast procedures reported in literature, confirming the generalizability of our study cohort [25–27]. In addition, our results are consistent with the findings of an earlier randomized controlled trial performed by our research group comparing the use of this short sodium bicarbonate regime to periprocedural saline hydration in patients with CKD undergoing intravenous contrast enhanced-computed tomography (8).

Some aspects of our study warrant comment. First, the majority of patients were randomized after the logistic arrangements for hospitalization planned for preventive hydration based on the use of standard saline hydration had been made. In those patients, the duration of hospitalization prior to the contrast procedure was not adjusted to the randomized treatment. As a result, healthcare cost savings associated with the use of sodium bicarbonate in our study might be underestimated. Second, our study was powered on an increase in serum creatinine and not on the risk of CI-AKI. We have chosen this primary endpoint for the following reasons: CI-AKI is a relatively rare event, which as a consequence would require a very large sample size in a trial with a non-inferiority design. Additionally, the definition of CI-AKI is often debated [1,20]. Moreover, the use of an increase in serum creatinine as a primary outcome allowed us to study subclinical changes in serum creatinine, that have been associated with mortality in other settings such as cardiac surgery [28,29]. Also, several other studies on the prevention of CI-AKI used a relative increase in serum creatinine as their primary outcome [8,24,30–36]. Third, 3/23 CI-AKI patients were lost for the endpoint of recovery of renal function. However, as the number of patients loss to follow-up was comparable for both randomization arms (1 in the sodium bicarbonate and 2 in the saline group), it is unlikely that this would have influenced the relative risk of renal function recovery comparing sodium bicarbonate with saline hydration. Fourth, our cost analysis was based on the Dutch healthcare system. The health economic impact might be less evident in countries where (saline) hydration is performed in day care in all patients with CKD or in countries where the sodium bicarbonate solution is not commercially available. Fifth, there was some imbalance in the type of contrast procedure between randomization arms. However, results on the primary outcome were consistent in a sensitivity analysis correcting for kind of contrast procedure (mean difference in relative serum creatinine increase between study arms 2.0%, 95% -1.2 to 5.1%). Sixth, based on the design of our study, we were unable to assess whether smaller volumes of saline (comparable with the sodium bicarbonate regime) would also be non-inferior to peri-procedural saline hydration. Additionally, we didn’t register patients that were ineligible for study participation.

In summary, this study indicates that a simple hydration regime using sodium bicarbonate administered 1 hour prior to elective cardiovascular diagnostic or interventional procedures requiring intra-arterial contrast administration is non-inferior to periprocedural saline hydration in patients with CKD. The use of this brief hydration protocol results in considerable healthcare cost savings. Further research is needed to study whether this short sodium bicarbonate regime can also be used in an emergency setting such as primary percutaneous coronary interventions, where the risk of (CI-) AKI is considered higher.

## Supporting information

S1 Fig Absolute serum creatinine values. Randomisation group 1 = sodium bicarbonate, randomisation group 2 = saline.
(DOCX)

S2 Fig eGFR values calculated using the MDRD-formula in patients with CI-AKI during follow-up. (DOCX)

S1 Supplemental Material List of participating hospitals. (DOCX)

S1 Consort Checklist (PDF)

S1 Trial Protocol (DOC)

S1 Dataset Consort 2010 checklist_Helios.pdf. (SAV)
