# Supplementary figures and images for: Randomized trial of one-hour sodium bicarbonate vs standard periprocedural saline hydration in chronic kidney disease patients undergoing cardiovascular contrast procedures
Source: PLoS One. 2018 Feb 8;13(2):e0189372. doi: 10.1371/journal.pone.0189372 (PMC5805164; doi:10.1371/journal.pone.0189372)

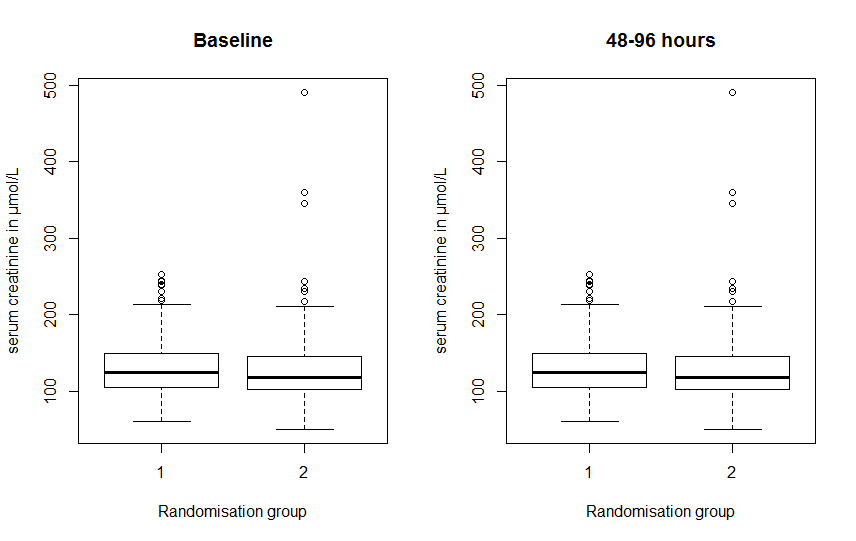

Supplement: S1 Fig — Randomisation group 1 = sodium bicarbonate, randomisation group 2 = saline. (DOCX) [file pone.0189372.s001.docx]

**
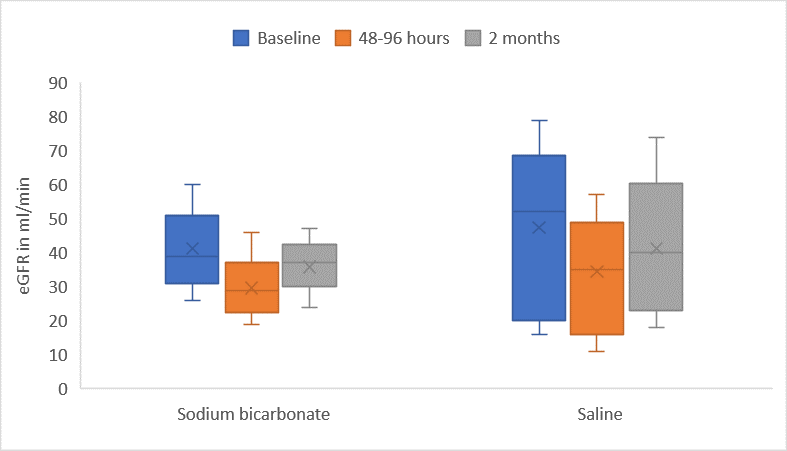
**

Supplement: S2 Fig — (DOCX) [file pone.0189372.s002.docx]
