# Supplementary material for: Randomized trial of one-hour sodium bicarbonate vs standard periprocedural saline hydration in chronic kidney disease patients undergoing cardiovascular contrast procedures
Source: PLoS One. 2018 Feb 8;13(2):e0189372. doi: 10.1371/journal.pone.0189372 (PMC5805164; doi:10.1371/journal.pone.0189372)
Supplement: S1 Supplemental Material — (DOCX) [file pone.0189372.s003.docx]

**Supplemental material**

**List of participating hospitals (all located in the Netherlands):**

- Leiden University Medical Center, Leiden. 57 inclusions
- St. Antonius Hospital, Nieuwegein. 142 inclusions
- Haga Teaching Hospital, The Hague. 57 inclusions
- Bronovo Hospital, The Hague. 43 inclusions
- St. Lucas Andreas Hospital, Amsterdam. 7 inclusions
- Onze Lieve Vrouwe Gasthuis, Amsterdam. 13 inclusions
- Maasstad Hospital, Rotterdam. 26 inclusions
