# Supplementary material for: Randomized trial of one-hour sodium bicarbonate vs standard periprocedural saline hydration in chronic kidney disease patients undergoing cardiovascular contrast procedures
Source: PLoS One. 2018 Feb 8;13(2):e0189372. doi: 10.1371/journal.pone.0189372 (PMC5805164; doi:10.1371/journal.pone.0189372)
Supplement: S1 Trial Protocol — (DOC) [file pone.0189372.s005.doc]

# Natriumbicarbonaat versus Natriumchloride ter preventie van contrastnefropathie na intra-arteriële contrasttoediening.

# Een gerandomiseerde non-inferiority studie

*De Helios studie*

Drs. J. Kooiman (1)

Dr. M.V. Huisman (1)

Dr. Y.W.J. Sijpkens (2)

(1) Afdeling Algemeen Interne Geneeskunde-Endocrinologie, LUMC

(2)Afdeling Interne Geneeskunde, Bronovo ziekenhuis, Den Haag

Correspondentieadres:

[J.Kooiman@lumc.nl](mailto:J.Kooiman@lumc.nl)

Post zone C4-70

Postbus 9600 2300 RC

# LUMC Leiden

# Aan dit protocol werkten mee:

*J. Kooiman*

*M.V. Huisman*

*Y.W.J. Sijpkens*

*C.S.P. van Rijswijk*

*A.J. Rabelink*

# Participerende ziekenhuizen

LUMC, Leiden – Dr. M.V. Huisman, afdeling Algemene Interne Geneeskunde - Endocrinologie

Bronovo Ziekenhuis, Den Haag – Dr. Y.W.J. Sijpkens, afdeling Interne Geneeskunde

HAGA Ziekenhuis, Den Haag – Dr. CJ van Rooden, afdeling Radiologie

St. Antonius Ziekenhuis, Nieuwegein – dr. J.P.P.M. de Vries, afdeling Vaatchirurgie

Sint Lucas Andreas Ziekenhuis, Amsterdam – Dr. H.F.H. Brulez, afdeling Nefrologie

Onze Lieve Vrouwe Gasthuis, Amsterdam – Dr. A.C. Vahl, afdeling Vaatchirurgie

Maasstad Ziekenhuis – Dr. I.J.A.M. Verberk-Jonkers, afdeling Nefrologie

**Inhoudsopgave**

*Synopsis 3*

*Achtergrond 5*

*Doel van het onderzoek 9*

*Vraagstelling 9*

*Ontwerp 10*

*Opzet 10*

*Patiënten 10*

*Methode 11*

*Bloed- en urineafname 12*

*Groepsgrootte en haalbaarheid 13*

*Duur en omvang van het onderzoek 13*

*Statistische analyse 14*

*Doelmatigheidsanalyse 14*

*Ethische overwegingen 15*

*Administratieve procedures 15*

*Onafhankelijk arts 15*

*Verzekering 15*

*Literatuur 16*

**Synopsis** Helios studie

***Introductie:*** Contrastnefropathie (CIN) kan optreden na toediening van jodiumhoudend contrastvloeistof ten behoeve van radiologisch onderzoek. Patiënten met een verminderde nierfunctie, vooral in combinatie met diabetes mellitus, hebben een verhoogd risico op het krijgen van CIN.

De CBO richtlijn adviseert daarom om risicopatiënten 4-12 uur voor en 4-12 uur na het onderzoek te behandelen met een natriumchloride (NaCl) infuus, afhankelijk van de cardiale conditie van de patiënt. Gevolg hiervan is dat patiënten die een radiologisch onderzoek met intra-arteriële contrasttoediening ondergaan en een indicatie hebben tot hydratie twee nachten opgenomen moeten worden. Een alternatief is een korte voorbehandeling met natriumbicarbonaat. Onderzoek in patiëntengroepen die hartkatheterisatie ondergingen liet zien dat deze behandeling gelijkwaardig is aan die met NaCl. Er is geen algemene richtlijn over de exacte uitvoering van hydratie met natriumbicarbonaat.

Na het ontstaan van contrastnefropathie herstelt de nierfunctie in vrijwel alle gevallen binnen twee maanden.
De CBO richtlijn wordt in klinieken ervaren als omslachtig in de uitvoering.

***Doel:*** Evaluatie van het optreden van CIN na diagnostiek en vasculaire ingrepen met intra-arteriële contrasttoediening van jodiumhoudende contrastvloeistof in hoeveelheden van 70 - 150 ml met een korte voorbehandeling met natriumbicarbonaat in vergelijking met het huidige beleid met NaCl.

***Studie opzet:*** Prospectief multi-center gerandomiseerd ‘non-inferiority’ onderzoek.

***Patiënten en methode:*** Patiënten die intra-arteriële contrasttoediening ondergaan met jodiumhoudende contrastmiddelen in hoeveelheden van 100 - 150 ml en een estimated glomerular filtration rate (eGFR) < 45 ml/min of eGFR < 60 ml/min in combinatie met diabetes mellitus (DM) of patiënten met een indicatie voor pre- en posthydratie volgens het ziekenhuisprotocol van het desbetreffend deelnemend centrum, worden gevraagd deel te nemen aan deze studie. In totaal zijn er 346 patiënten nodig. De patiënten worden via randomisatie verdeeld over twee studie armen.

- Groep 1: natriumbicarbonaat 1,4% 250 ml per infuus 1 uur voor contrasttoediening.
- Groep 2: NaCl 0,9% 1000 ml 3-12 uur vóór en na arteriële contrasttoediening, afhankelijk van de cardiale conditie van de patiënt.

***Diagnostische testen:*** De nierfunctie en markers voor nierschade worden gemeten voorafgaand, 4 uur, 2-4 dagen en op indicatie 2 maanden na arteriële contrasttoediening.

***Studie eindpunten:***

*Primair:*

1. Gemiddelde stijging in serumcreatinine 2-4 dagen na contrasttoediening.

*Secundair:*

1. CIN gedefinieerd als een stijging in serum creatinine > 25% of > 44 µmol/l na 2-4 dagen;
2. CIN na twee maanden;
3. Aantal patiënten dat een indicatie tot dialyse ontwikkelt;
4. Manifest hartfalen ten gevolgen van pre- en posthydratie met indicatie voor het geven van diuretica;
5. Chronisch nierfunctieverlies 1 jaar na contrasttoediening gedefinieerd als een stijging in serum creatinine > 25% of > 44 µmol/l.;
6. Aantal ligdagen en opnames 2 maanden en tot 1 jaar na contrasttoediening.

**Achtergrond**

Intra-arteriële toedieningen met jodiumhoudend contrastmiddelen vindt plaats bij diagnostische en therapeutische endovasculaire procedures zoals digitale subtractie angiografie (DSA), percutane transluminale angioplastie (PTA), endovasculaire stent plaatsing bij abdominale of thoracale aneurysmata (EVAR) en coiling van intracraniele aneurysmata.

Een DSA-onderzoek vindt plaats voor het diagnosticeren van onder andere stenosen, occlusies, trombose, vaatanomalies, aneurysmata en bloedingen. Het onderzoek kan ook worden verricht om de anatomie in kaart te brengen voorafgaand aan een (vaatchirurgische) ingreep. Wanneer specifiek naar de coronairarteriën gekeken wordt spreekt men van een hartkatheterisatie.

Een PTA wordt uitgevoerd bij patiënten met een vernauwing of occlusie van een arterie in de bovenste of onderste extremiteit. Bij een PTA kan worden volstaan met een dotter procedure echter in bepaalde gevallen kan aanvullend een stent worden geplaatst.

Bij een EVAR-procedure wordt een stent in de thoracale of abdominale aorta geplaatst om een aneurysma uit te schakelen en de kans op een ruptuur te voorkomen.

Bij coiling van intracraniële aneurysmata worden meerdere coils in het aneurysmata geplaatst waardoor de kans op een intracraniële bloeding (of hernieuwde bloeding) wordt gereduceerd.

Gedurende bovengenoemde procedures wordt gemiddeld 70-150 ml non-ionische laag osmolair contrastmiddel intraarterieel toegediend, (Ultravist concentratie 300 mgI/ml). De hoeveelheid contrastmiddel is afhankelijk van de duur en soort procedure en het lichaamsgewicht van de patiënt. Patiënten lopen hierbij risico op het ontstaan van contrastnefropathie (CIN). Er wordt gesproken van CIN bij een stijging in creatinine van minimaal 25% of van 44 µmol/l in de 3 tot 5 dagen na contrasttoediening (1). Patiënten met een al bestaande nierinsufficiëntie hebben een verhoogd risico op het ontstaan van contrastnefropathie (2).

Een voorbehandeling met natriumchloride -0,9% 1ml/uur/kg lichaamsgewicht gedurende 4 tot 12 uur- reduceert het risico op contrastnefropathie (3, 4). De toedieningtijd van dit infuus is verlengd als de patiënt bekend is met onderliggend hartfalen. Het mechanisme waarlangs de bescherming van de pre- en posthydratie verloopt, heeft vooral te maken met het voorkomen van ondervulling ten tijde van het onderzoek.

Volgens de richtlijn van het CBO *voorzorgsmaatregelen bij jodiumhoudende contrastmiddelen (2007)* moeten patiënten met een eGFR < 45 ml/min of een eGFR < 60 ml/min in combinatie met diabetes mellitus zowel een voorbehandeling als een nabehandeling krijgen met 0,9 % natriumchloride 1 ml/uur/kg lichaamsgewicht elk gedurende 4 tot 12 uur (1). Patiënten moeten hierdoor 1 tot 3 dagen opgenomen worden voor een behandeling met intra-arteriele contrasttoediening. Doordat de meeste patiënten die een EVAR- of PTA-procedure ondergaan naast vasculair ook cardiaal belast zijn komt dit meestal neer op een opname van 3 dagen waarbij het infuus langzaam toegediend wordt om hartfalen te voorkomen.

Om te bepalen welke patiënten gescreend moeten worden op een indicatie voor hydratie zijn indicaties gesteld door de CBO richtlijn ‘Voorzorgsmaatregelen bij jodiumhoudende contrastmiddelen’. De eGFR dient bepaald te worden indien patiënten voldoen aan minimaal één van de volgende criteria (1):

- Leeftijd > 60 jaar;
- Diabetes mellitus;
- Hart- en vaatlijden;
- Hypertensie;
- Urologische of nefrologische voorgeschiedenis;
- M. Kahler of M. Waldenström met uitscheiding van lichte ketens in urine;
- Gebruik van medicatie zoals diuretica en NSAID’s, metformine en aminoglycosiden.

Veel ziekenhuizen hebben voor deze screening een zogenaamde *contrastpolikliniek* opgezet.

Om de nierfunctie te bepalen en daarmee de indicatie tot voorbehandeling met hydratie, wordt de eGFR geschat met behulp van de *Modification of Diet in Renal Disease* (MDRD) formule (5, 6).

Een belangrijk gegeven is dat meer dan 25% van de gezonde 65 jarige mannen en 50% van de gezonde 65 jarige vrouwen een eGFR < 60 ml/min heeft (7). Een eGFR 50 – 60 ml/min bij oudere patiënten hoeft niet altijd van klinisch belang te zijn (8). Dit is meestal geen uiting van nierziekte maar van een lager aantal aangelegde nefronen en fysiologisch nierfunctieverlies dat gepaard gaat met veroudering. Hierdoor wordt de groep patiënten die op dit moment volgens het CBO een indicatie heeft voor hydratie erg groot. Het is belastend om die grote groep 1 tot 3 dagen op te nemen voor de contrasttoediening, voor zowel de patiënt als de afdeling en is misschien niet in alle gevallen noodzakelijk. Dit gaat ten kosten aan de uitvoerbaarheid van de richtlijn (8). De CBO richtlijn wordt dan ook in de kliniek ervaren als omslachtig in de uitvoering en is belastend voor de patiënt.

Een alternatief voor een hydratieregime met NaCl is een infuus met natriumbicarbonaat 1.4% (3 ml/uur/kg lichaamsgewicht). Dit infuus wordt 1 uur voor en 4-6 uur na de contrastinjectie toegediend. In verschillende onderzoeken is aangetoond dat deze voorbehandeling met natriumbicarbonaat gelijkwaardig is aan de huidige behandeling met natriumchloride (9 - 17).

De beschermende werking van natriumbicarbonaat wordt op twee manieren verklaard. Ten eerste zorgt het voor volume expansie. Wanneer de afname in contrastnefropathie wordt uitgezet tegen het verschil in de toename van extracellulair volume dan blijkt er een lineaire correlatie (1). Het preventieve effect van hydratie wordt dus mede bepaald door de hoeveelheid zoutoplossing (1). Ten tweede zorgt natriumbicarbonaat voor alkalinisatie van vrije zuurstof radicalen. Dit beschermt de tubuluscel tegen chemische schade (1).

Het natriumbicarbonaat regime zorgt ervoor dat patiënten nog maar 1 dag opgenomen hoeven te worden voor het uitvoeren van diagnostiek of therapie met toediening van intra-arteriëel contrast. Echter de logistiek rond de intra-arteriele contrasttoediening is door de posthydratie van 6 uur nog steeds niet optimaal. De intra-arteriele contrastprocedures voor hoogrisicopatiënten moeten ook met dit hydratiebeleid op een vastgesteld tijdstip ingepland worden zodat er nog voldoende tijd is voor de posthydratie alvorens een patiënt naar huis toe kan. Dit bemoeilijkt en vertraagt de inplanning van contrastonderzoek voor patiënten met een verhoogd risico op CIN.

Het effect van de prehydratie berust grotendeels op het voorkomen van dehydratie en ondervulling ten tijde van de contrasttoediening (18). De meerwaarde van posthydratie is nooit onderzocht en er is geen rationale voor een toegevoegde waarde van de posthydratie ter bescherming van de nier. Op grond hiervan is het zeer aannemelijk dat 250 ml natriumbicarbonaat (de hoeveelheid gebruikt voor de prehydratie) de nier voldoende beschermt tegen CIN.

Mede ingegeven door bovenstaande overwegingen wordt in veel ziekenhuizen een hydratieregime met natriumbicarbonaat zonder posthydratie toegepast bij spoedonderzoek met intraveneus contrast. Dit hydratieregime wordt als veilig en effectief beoordeeld. Uit onlangs afgesloten en nog niet gepubliceerd retrospectief onderzoek, uitgevoerd in het Bronovo ziekenhuis blijkt dat bij intraveneuze contrasttoediening de incidentie van contrastnefropathie na een hydratieregime bestaande uit alleen een prehydratie met natriumbicarbonaat 4,5% bedraagt. De incidentie contrastnefropathie werd in dit onderzoek gedefinieerd als een stijging in serum creatinine van meer dan 25 % of een stijging van meer dan 44 µmol/l. In een vergelijkbaar onderzoek bleek de incidentie van contrastnefropathie in het LUMC, waar NaCl pre- en posthydratie gegeven wordt, 8.9% te bedragen(19). Na 1 maand voldeed nog 0.04% van de patiënten aan de definitie van contrastnefropathie (19). De nierfunctie herstelt dus in het overgrote deel van de patiënten (19). Het absolute risico op duurzaam en klinisch relevant nierfunctieverlies door contrastnefropathie lijkt dan ook zeer beperkt (1, 17). Voorheen werd aangenomen dat het risico op CIN hoger is na intra-arteriële contrasttoediening dan na waneer dit contrast intraveneus wordt toegediend. Tot op heden is daar geen wetenschappelijk bewijs voor.

Uit een pilot studie uitgevoerd in het LUMC waarbij 40 patiënten zowel intra-arterieel als intraveneus contrast toegediend hebben gekregen bleek CIN niet vaker voor te komen na intra-arteriële contrasttoediening. CIN kwam in 2/40 patiënten (5%) voor na intra-arteriële contrasttoediening en in 3/40 patiënten (7.5%) na CT-scan met intraveneuze contrasttoediening.

Een kort natriumbicarbonaat regime heeft grote voordelen boven NaCl dat in twee dagen gegeven wordt:

- Een kort hydratieregime met natriumbicarbonaat heeft een groot logistiek en economisch voordeel. De interventieradiologische procedure kan elk moment van de dag plaats vinden, ook wanneer dit niet lang van te voren aangevraagd is. Poliklinische patiënten kunnen na het onderzoek direct terug naar huis en hoeven niet opgenomen te worden voor posthydratie. Doordat dit hydratieregime eenvoudig is en weinig tijd kost is het goed implementeerbaar in de dagelijkse praktijk, waarin steeds meer interventieradiologische procedures toegepast worden en steeds meer mensen een indicatie hebben tot preventieve maatregelingen bij contrasttoediening.
- Het risico op overvulling bij cardiaal belaste patiënten is vele malen kleiner na het toedienen van 250 ml natriumbicarbonaat ten opzichte van 2 liter NaCl. Deze overvulling zou behandeld moeten worden en leidt tot verder uitstel van het contrastonderzoek.

Er is weinig bekend over het effect van CIN op het behoud van nierfunctie, morbiditeit en mortaliteit op langer termijn. Door één jaar na contrasttoediening de nierfunctiebepaling te herhalen en de verderop beschreven doelmatigheidsanalyse uit te voeren, wordt geanalyseerd in hoeverre CIN en een stijging in NGAL of KIM-1 verspellend zijn voor chronisch nierfunctie verlies, morbiditeit en mortaliteit na het toedienen van contrastmiddelen.

Door middel van deze gerandomiseerde studie wordt de effectiviteit van een natriumbicarbonaatregime zonder posthydratie onderzocht. Het is zeer waarschijnlijk dat het weglaten van de posthydratie bij een natriumbicarbonaatregime weinig effect zal hebben op de nierfunctie en het optreden van contrastnefropathie. Wanneer dit hydratieregime non-inferior blijkt te zijn aan het bestaande hydratieregime met NaCl zal dit grote invloed hebben op de dagelijkse praktijk en de logistiek rond contrastonderzoek praktischer vereenvoudigen.

**Doel van het onderzoek**

Doel van het onderzoek is het aantonen of een korte voorbehandeling met natriumbicarbonaat net zo veilig is als de bestaande voor- en nabehandeling met natriumchloride.

# Vraagstelling:

Is de gemiddelde stijging in serumcreatinine na een korte voorbehandeling met natriumbicarbonaat niet significant verschillend ten opzichte van de huidige voor- en nabehandeling met natriumchloride bij patiënten die een interventieradiologisch onderzoek ondergaan met een arteriële contrastbelasting tussen 70-150 ml in concentraties van maximaal 400 mg/ml?

**Ontwerp van het onderzoek**

De studie is opgezet als een prospectief non-inferiority multicenter gerandomiseerd onderzoek.

*Primair eindpunt*

- Gemiddelde stijging in serumcreatinine 2-4 dagen na contrasttoediening.

*Secundaire eindpunten*

- Stijging in serumcreatinine >25% of > 44 µmol/L, 2 tot 4 dagen na

contrasttoediening;

- Stijging in serumcreatinine >25% of > 44 µmol/L, 2 maanden na contrasttoediening;
- Aantal patiënten dat een indicatie tot dialyse ontwikkelt;
- Aantal patiënten met manifest hartfalen binnen 24 na start prehydratie.
- Chronisch nierfunctieverlies één jaar na contrasttoediening gedefinieerd als een stijging in serum creatinine > 25% of > 44 µmol/l.
- Aantal ligdagen en opnames twee maanden en tot één jaar na contrasttoediening.

**Opzet van het Onderzoek**

Selectiecriteria

Inclusiecriteria

- Patiënten met een eGFR < 45 ml/min;
- Patiënten met een eGRF < 60 ml/min en diabetes mellitus;
- Patiënten met een indicatie voor pre- en posthydratie volgens het ziekenhuisprotocol van het desbetreffend deelnemend centrum;
- Informed consent.

Exclusiecriteria

- Patiënten jonger dan 18 jaar;
- Patiënten die < 7 dagen geleden beeldvormend onderzoek met contrastvloeistof hebben ondergaan;
- Patiënten met acuut nierfalen;
- Zwangerschap;
- M. Kahler;
- Bekende allergie voor jodiumhoudende contrastmiddelen.

**Methode**

*Algemeen*

Van alle patiënten die PTA, EVAR, stenting, neuro-coiling of DSA ondergaan wordt tevoren het creatinine en daarmee de eGFR bepaald. Tevens worden de volgende gegevens van de patiënt geregistreerd:

- Leeftijd;
- Geslacht;
- Afro-Americaanse etniciteit;
- Lengte;
- Gewicht voor start prehydratie;
- Bloeddruk;
- Pols;
- Reden voor intra-arteriële contrasttoediening (perifeer vaatlijden, aneurysma, e.d.);
- Nierziekte of urologische ziekte in de voorgeschiedenis;
- Rookgedrag, in aantal packyears;
- Gebruik van medicatie en dosering;
- Stop medicatie de intra-arteriële contrasttoeding;
- Soort en dosis contrastmiddel;
- Diabetes mellitus, type en aantal jaren;
- Tijd tussen creatininemeting de contrasttoediening en 3-5 dagen na toediening;
- Hemoglobine, creatinine, ureum, urinezuur, fosfaat;
- eGFR voor toediening contrastvloeistof;
- Urine albumine/creatinine ratio;
- Urine pH en NGAL.

Patiënten met een GFR < 45 ml/min, een GFR 45- 60 ml/min en diabetes mellitus of patiënten met een indicatie voor pre- en posthydratie volgens het ziekenhuisprotocol van het desbetreffend deelnemend centrum worden na toestemming geïncludeerd.

De geïncludeerde patiënten worden na randomisatie ingedeeld in een van de volgende twee groepen:

- Groep 1: prehydratie met natriumbicarbonaat 1.4% 250 mL 1 uur voor de contrasttoediening. Vier uur na contrasttoediening wordt bloed en urine afgenomen waarna deze groep meteen naar huis kan.
- Groep 2: ondergaat zowel pre- als posthydratie volgens het huidige beleid met 1000 ml natriumchloride 0,9% in 3-12 uur afhankelijk van de cardiale conditie van de patiënt. Vier uur na contrasttoediening wordt bloed en urine afgenomen waarna patiënten uit deze groep blijven voor de posthydratie.

*Urine- en bloedafname*

- Voorafgaand aan de contrasttoediening wordt bloed en urine afgenomen voor bepaling van markers van nierfunctie, nierschade en diabetes mellitus: hemoglobuline, ureum, creatinine, de eGFR door middel van de MDRD, urinezuur, fosfaat, NGAL, Cystatine-C en de albumine/creatinine ratio, KIM-1 pH in de urine. Het bepalen van serumcreatinine behoort tot standaard zorg. Van elke patiënt wordt een buisje bloed opgeslagen voor de nabepaling van Cystatine-C en NGAL.
- Na 4 uur de contrasttoediening wordt van alle patiënten een portie urine gevraagd om daarin het pH, KIM-1 en urine NGAL te bepalen en wordt bloed afgenomen voor de bepaling van het serum NGAL. Om te bepalen hoeveel vocht patiënten vasthouden worden alle patiënten 4 uur na contrasttoediening nogmaals gewogen.
- 2-4 dagen na de contrasttoediening komen alle patiënten terug voor bloedafname naar het ziekenhuis. Indien de patiënt nog opgenomen is na 3 dagen wordt de bloedafname door de afdeling geregeld. Alle patiënten krijgen na inclusie een speciaal labformulier waarmee deze bepalingen aangevraagd kunnen worden. Patiënten die niet naar het ziekenhuis komen worden opgebeld. Eventueel wordt thuis bloed afgenomen. In het laboratorium wordt het volgende direct bepaald: plasma-creatinine, eGFR door middel van de MDRD, ureum, urinezuur, fosfaat en KIM-1 en albumine/creatinine ratio in de urine. Van elke patiënt wordt een buisje bloed opgeslagen voor de nabepaling van Cystatine-C en NGAL.
- Indien na 2-4 dagen CIN wordt vastgesteld zal de nierfunctie na 2 maanden gecontroleerd worden middels de volgende bepalingen: plasma-creatinine, eGFR door middel van de MDRD, cystatine C, ureum, urinezuur, fosfaat en albumine/creatinine ratio. Hierbij moet er 1 buisje bloed en urine opgeslagen worden voor de nabepaling van Cystatine-C en NGAL.
- Na 1 jaar wordt bij alle patiënten voor de laatste keer bloed en urine afgenomen. Hierin zullen dezelfde bepalingen als voorafgaand aan contrasttoediening herhaald worden.

**Groepsgrootte en haalbaarheid**

De studie is opgezet als een *non-inferiority* onderzoek. Aan hand van gegevens uit retrospectief onderzoek is gebleken dat na arteriële contrasttoediening het serumcreatinine gemiddeld -1.6 % stijgt bij een standaarddeviatie van 31 %. Gesteld wordt dat natriumbicarbonaat non-inferior is aan natriumchloride wanneer het verschil in gemiddelde serumcreatininestijging per groep kleiner is dan 15%. Om een verschil in gemiddelde serumcreatininestijging van minimaal 15% waar te nemen bij een standaarddeviatie van 31 % zijn ongeveer 150 patiënten per studiearm nodig bij een power van 80%. Dit is de power onder aanname van een daadwerkelijk verschil in stijging van 5%. Rekening gehouden met een lost to follow-up van 15% zullen in totaal 173 patiënten per arm geincludeerd worden. Dit leidt tot een totale studiepopulatie van 346 patiënten.

**Duur en omvang van het onderzoek**

In het LUMC ondergaan per jaar 42 patiënten met een indicatie tot hydratie (eGFR < 45 ml/min of eGFR < 60 ml/min en diabetes mellitus ) PTA, 7 EVAR, 60 DSA, 18 stent plaatsing en 25 neuro-coiling procedures.

Hiermee ligt het totaal op 152 per jaar. Rekening houdend met het risico dat 20% van de patiënten deelname weigert, komt dit neer op 120 patiënten per jaar, per ziekenhuis.

De inclusie zal maximaal 2.5 jaar duren bij deelnamen van 3 ziekenhuizen, inclusief één jaar follow-up.

# Statistische analyse

- Berekenen van de gemiddelde stijging in serumcreatinine 2-4 dagen na contrasttoediening.  Het verschil tussen de beide armen wordt geschat, samen met een 95% betrouwbaarheids interval voor dit verschil.
- Aan hand van de analyse op de gemiddelde stijging in serumcreatinine 2-4 dagen na contrasttoediening, wordt het effect van beide hydratieregimes op het voorkomen van contrastnefropathie geanalyseerd. Dit gebeurt op basis van zowel de data per arm als het geschatte verschil tussen de armen en de standaard deviatie daarvan. Op basis van deze effectmaten wordt teruggerekend in welk percentage patiënten de stijging zodanig is dat er sprake is van contrastnefropathie. Voor dit percentage wordt vervolgens weer een betrouwbaarheids interval geschat (derhalve percentiel-schatting op basis van de geobserveerde data). Dit gebeurt per arm. Tot slot wordt het verschil in percentage contrastnefropathie berekend als verschil tussen deze percentages. Omdat de groepen onafhankelijk zijn, volgt de geschatte standaarddeviatie van het verschil in percentages contrastnefropathie rechtstreeks uit de standaarddeviaties binnen de beide armen.
- Bereken van de Odds Ratio CIN voor het hydratieschema met natriumbicarbonaat ten opzichte van het huidige regime met natriumchloride.
- Berekenen van de gemiddelde absolute daling in eGFR voor beide groepen.
- Het berekenen van risicofactoren voor contrastnefropathie voor beide groepen.
- Berekenen van de Odds Ratio CIN voor een stijging in NGAL.
- Berekenen van de Odds Ratio CIN voor een stijging in cystatine-C.

**Doelmatigheidsanalyse**

Om te oordelen of de behandeling met natriumbicarbonaat leidt tot een stijging in het aantal ligdagen dan wel opnames en poliklinische contacten tot 2 maanden en tot één jaar na de contrasttoediening als gevolge van een mogelijk minimaal verhoogd risico op CIN, zal deze informatie voor alle patiënten uit het ziekenhuissysteem worden opgezocht.

In de kostenanalyse zullen naast de primaire behandeling (hydratie met bijbehorende opnameduur) ook andere ziekenhuiskosten gedurende de initiële 2 maanden worden meegenomen (internist- en nefroloog consulten en ziekenhuis opnameduur). In een kosteneffectiviteitanalyse zullen deze kosten worden gerelateerd aan het risico op CIN (kosten per voorkomen CIN).

**Ethische overwegingen**

*Informed consent*

Informed consent wordt aan elke patiënt gevraagd nadat het onderzoek uitvoerig is uitgelegd en mogelijke gevolgen ten opzichte van de gangbare diagnostische praktijk verhelderd zijn. Elke patiënt dient zijn toestemming schriftelijk vast te leggen op het formulier zoals bijgevoegd (zie bijlage). Intrekking van toestemming door de patiënt is op ieder tijdstip van het onderzoek mogelijk ongeacht de redenen hiervoor en zonder nadelige gevolgen voor de patiënt.

# Administratieve procedures

De onderzoeker verzekert de patiënt dat anonimiteit voor derden gewaarborgd blijft. Dit geschiedt doordat elke patiënt slechts met een studienummer en zijn initialen vermeld wordt op de studieformulieren.

# Onafhankelijk arts

Onafhankelijk arts voor deze studie is Dr. J Fogteloo, internist, bereikbaar op telefoonnummer: 071-5262085

**Verzekering**

# Voor de proefpersonen is een verzekering afgesloten.

**Literatuur**

1. Kwaliteitsinstituut voor de Gezondheidszorg CBO. Richtlijn Voorzorgsmaatregelen bij jodiumhoudende contrastmiddelen, Nederlandse Vereniging voor Radiologie, 2007
2. Persson PB, Hansell P, Liss P. Pathophysiology of contrast medium-induced nephropathy. *Kidney Int.* 2005 Jul; 68(1):14-22.
3. Mueller C, Buerkle G, Heinz J et al. Prevention of contrast media–associated nephropathy, *Arch Intern Med.* 2002;162:329-336.
4. Solomon R, Werner C, Mann D, et al. Effects of saline, mannitol, and furosemide on acute decreases in renal function induced by radiocontrast agents. *The* *New England Journal of Medicine* 1994; 331:1416-1420
5. Levey AS, Coresch J, Greene T, et al. Using standardized serum creatinine values in the modification of diet in renal disease study equation for estimating glomerular filtration rate. *Ann Intern Medicine* 2006;145:247-54
6. Tanaka A, Suemaru K, Araki H. A new Approach for Evaluating Renal Function and Its Practical Application *J Pharmacol Sci* 2007; 105, 1-5
7. Elsevier MM, Verpooten GA, DeBroe ME, et al. Interpretation of creatinine clearance *The Lancet* 1987 February 21; 457
8. O’Hare AM, Bertenthal D, Covinsky KE, et al. Mortality risk stratification in chronic kidney disease: one size for all ages? *J Am Soc Nephrol.* 2006 March; 17 (3):846-853. Epub 2006 Feb 1.
9. Merten GJ; Burgess WP; Gray LV, et al. Prevention of contrast-induced nephropathy with sodium bicarbonate: a randomized controlled trial. *JAMA* 2004 May 19;291(19):2328-34.
10. Briguori C; Airoldi F; D'Andrea D, et al. Renal Insufficiency Following Contrast Media Administration Trial (REMEDIAL): a randomized comparison of 3 preventive strategies. *Circulation* 2007 Mar 13;115(10):1211-7. Epub 2007 Feb 19.
11. Recio-Mayoral A; Chaparro M; Prado B, et al. The reno-protective effect of hydration with sodium bicarbonate plus N-acetylcysteine in patients undergoing emergency percutaneous coronary intervention: the RENO Study. *J Am Coll Cardiol*. 2007 Mar 27;49(12):1283-8. Epub 2007 Mar 12
12. Ozcan EE; Guneri S; Akdeniz B, et al. Sodium bicarbonate, N-acetylcysteine, and saline for prevention of radiocontrast-induced nephropathy. A comparison of 3 regimens for protecting contrast-induced nephropathy in patients undergoing coronary procedures. A single-center prospective controlled trial. *Am Heart J.* 2007 Sep;154(3):539-44
13. Brar SS; Shen AY; Jorgensen MB, et al. Sodium bicarbonate vs sodium chloride for the prevention of contrast medium-induced nephropathy in patients undergoing coronary angiography: a randomized trial. *JAMA* 2008 Sep 3;300(9):1038-46
14. Maioli M; Toso A; Leoncini M, et al. Sodium bicarbonate versus saline for the prevention of contrast-induced nephropathy in patients with renal dysfunction undergoing coronary angiography or intervention. *J Am Coll Cardiol*. 2008 Aug 19;52(8):599-604
15. Navaneethan SD, Singh S, Appasamy S, et al. Sodium Bicarbonate Therapy for Prevention of Contras-Induced Nephropathy: A systematic Review and Meta-analysis *American Journal of Kidney Disease* 2009 Apr;53(4):617-27
16. Meier P, Ko DT, Tamura A, et al. Sodium bicarbonate-based hydration prevents contrast-induced nephropathy: a meta-analysis *BCM Medicine* 2009 7:23
17. Kanbay M, Covic A, Coca SG, et al. Sodium bicarbonbate for the prevention of contrast-induced nephropathy: a meta-analysis of 17 randomized trials Int Urol Nephrol 2009 41:617-627
18. McCullough PA. Radiocontrast-induced Acute Kidney Injury. Nephron Physiol 2008; 109: 61-72
19. Kooiman J, Klok FA, Huisman MV, et al. Contrast-induced nephropathy following CT-angiography for suspected acute pulmonary embolism. J Thromb Haemost. 2010 Feb;8(2):409-11.
